# Dopamine pathway gene variants may modulate cognitive performance in the DHS – Mind Study

**DOI:** 10.1002/brb3.446

**Published:** 2016-03-15

**Authors:** Susan E. Martelle, Laura M. Raffield, Nichole D. Palmer, Amanda J. Cox, Barry I. Freedman, Christina E. Hugenschmidt, Jeff D. Williamson, Don W. Bowden

**Affiliations:** ^1^ Department of Physiology and PharmacologyWake Forest School of MedicineWinston ‐ SalemNorth Carolina; ^2^ Center for Genomics and Personalized Medicine ResearchWake Forest School of MedicineWinston ‐ SalemNorth Carolina; ^3^ Molecular Basis of DiseaseGriffith UniversitySouthportBrisbaneQueenslandAustralia; ^4^ Department of Internal Medicine, NephrologyWake Forest School of MedicineWinston ‐ SalemNorth Carolina; ^5^ Department of Internal Medicine, Gerontology and Geriatric MedicineWake Forest School of MedicineWinston ‐ SalemNorth Carolina

**Keywords:** Cognition, dopamine, DAT1 VNTR, DOPA decarboxylase, polymorphism, type 2 diabetes

## Abstract

**Background:**

There is an established association between type 2 diabetes and accelerated cognitive decline. The exact mechanism linking type 2 diabetes and reduced cognitive function is less clear. The monoamine system, which is extensively involved in cognition, can be altered by type 2 diabetes status. Thus, this study hypothesized that sequence variants in genes linked to dopamine metabolism and associated pathways are associated with cognitive function as assessed by the Digit Symbol Substitution Task, the Modified Mini‐Mental State Examination, the Stroop Task, the Rey Auditory‐Verbal Learning Task, and the Controlled Oral Word Association Task for Phonemic and Semantic Fluency in the Diabetes Heart Study, a type 2 diabetes‐enriched familial cohort (*n* = 893).

**Methods:**

To determine the effects of candidate variants on cognitive performance, genetic association analyses were performed on the well‐documented variable number tandem repeat located in the 3' untranslated region of the dopamine transporter, as well as on single‐nucleotide polymorphisms covering genes in the dopaminergic pathway, the insulin signaling pathway, and the convergence of both. Next, polymorphisms in loci of interest with strong evidence for involvement in dopamine processing were extracted from genetic datasets available in a subset of the cohort (*n* = 572) derived from Affymetrix^®^ Genome‐Wide Human SNP Array 5.0 and 1000 Genomes imputation from this array.

**Results:**

The candidate gene analysis revealed one variant from the DOPA decarboxylase gene, rs10499695, to be associated with poorer performance on a subset of Rey Auditory‐Verbal Learning Task measuring retroactive interference (*P* = 0.001, *β *= −0.45). Secondary analysis of genome‐wide and imputed data uncovered another DOPA decarboxylase variant, rs62445903, also associated with retroactive interference (*P* = 7.21 × 10^−7^, *β *= 0.3). These data suggest a role for dopaminergic genes, specifically a gene involved in regulation of dopamine synthesis, in cognitive performance in type 2 diabetes.

## Introduction

Although cognitive decline is a part of normal aging, the rate of cognitive decline can be influenced by factors such as lifestyle, health, and genetics. Previous epidemiological and imaging studies have established an association between type 2 diabetes (T2D) and accelerated cognitive decline (Manschot et al. 2006; Luchsinger 2012; Palta et al. 2014; Ryan et al. 2014). In a review on the relationship between diabetes and cognition, Mayeda and colleagues postulate that insulin dysregulation and hyperglycemia are causal factors in neurodegeneration and cognitive decline (2015). However, the exact mechanism linking diabetes and poor cognitive performance remains unclear.

DA (Dopamine), a catecholamine neurotransmitter, plays a major role in learning and behavior by modulating neural activity in the prefrontal cortex (Clark and Noudoost 2014). DA has been implicated in disorders that influence cognitive performance such as ADHD (attention‐deficit/hyperactivity disorder, Genro et al. 2010; Sharma and Couture 2014) and Parkinson's disease (Kehagia et al. 2013; Ko et al. 2013). Several studies have examined the influence of genetic polymorphisms of genes from the dopaminergic pathway on cognitive performance. These studies have reported significant evidence of association with a range of cognitive measures (e.g., Savitz et al. 2006; Frank and Fossella 2011; Nemoda et al. 2011; Bowirrat et al. 2012; Störmer et al. 2012). One polymorphism that has received considerable attention is the variable number tandem repeat located in the 3' untranslated region of the DA transporter gene, (*DAT1* VNTR, rs28363170) which has been associated with several aspects of executive functioning (Brown et al. 2011; Wittmann et al. 2013; Gordon et al. 2015; Sambataro et al. 2015; Schneider et al. 2015).

Insulin insufficiency is a characteristic of individuals with T2D, where peripheral insulin resistance is not compensated for by adapting insulin secretion, thus resulting in chronic hyperglycemia (Wu et al. 2014). Beyond insulin's fundamental role in metabolic regulation (Plum et al. 2006), insulin dysregulation may also be centrally involved in DA homeostasis (Kleinridders et al. 2015). For example, insulin receptors are collocated on DA neurons in the substantia nigra (Henderson et al. 2000), a region involved in cognitive control (Boehler et al. 2011), decision‐making (Ding and Gold 2013), and reinforcement learning (de Berker and Rutledge 2014). Not insignificantly, pancreatic *β*‐cells co‐express the DA D3 receptor and transporter, *DRD3* and *DAT1* respectively, which are implicated in inhibitory control of glucose‐stimulated insulin release (Ustione and Piston 2012). Thus, it is reasonable to consider the impact of polymorphisms in the DA pathway in the pathogenesis of T2D complications, including cognitive decline. Based upon these observations, the purpose of this study was twofold: (1) to investigate the role of common and multi‐allelic genetic variants in cognitive performance; and (2) to perform a focused association analysis of dopaminergic pathway loci using genotyped and imputed data.

The majority of reports in the DA candidate gene literature contain relatively small sample sizes with modest coverage of the genes in question. Therefore, this study took advantage of an extensively genotyped familial cohort of T2D patients and related controls, the Diabetes Heart Study (DHS) – Mind, to investigate the role of sequence variants in genes linked to DA metabolism (e.g., *DDC*,* TH*,* DRD2*,* DRD3*, and *DAT1*) and associated pathways in cognitive function. We hypothesized that polymorphisms in these pathways were associated with performance on a cognitive battery in T2D‐affected individuals and unaffected siblings.

## Material and Methods

### Subjects

In all, 893 participants from 550 families of European ancestry (783 T2D‐affected; 110 controls) were recruited (Table 1). Recruitment and ascertainment for the study have been described elsewhere (Bowden et al. 2010; Hugenschmidt et al. 2013; Raffield et al. 2015). Briefly, siblings concordant for T2D, plus available nondiabetic siblings, were enrolled. T2D, confirmed by fasting glucose and glycated hemoglobin (HbA_1C_), was defined as occurring after the age of 35 years, without advanced renal insufficiency, and not initially treated exclusively by insulin therapy. The DHS – Mind sample is an ancillary study to the original DHS initiated in 2008 to examine the relationship between T2D and cognitive function (for a review of the DHS family of studies, see Bowden et al. 2010). The current analysis included a T2D enriched sample of 321 newly recruited T2D‐affected participants in addition to 573 individuals initially recruited in the original DHS investigation from 1998 to 2005.

**Table 1:** Demographic characteristics of the 893 DHS–Mind participants

	Mean (SD) or %	Median (range)
Demographic Information		
Age (years)	65.8 (9.7)	66.2 (37.7–93.2)
Gender (% female)	52.9	
BMI (kg/m^2^)	32.5 (6.7)	31.6 (14.6–59)
% smoking (current and prior)	54.6	
Hypertension (%)	86.9	
Self‐reported history of prior CVD (%)	34.8	
Type 2 diabetes		
% affected	87.7	
Duration (year)	15.4 (7.7)	13.8 (0.4–44)
Glucose (mg/dL)	147.6 (55)	135 (40–408)
Hemoglobin A1C (%)	7.3 (1.4)	7.3 (1.9–14.8)
Medication use (%)		
Antidiabetic	73.2	
Cholesterol‐lowering	52.3	
Antihypertensive	67.4	
Education (%)		
Less than high school	14.8	
High school	47.7	
Greater than high school	37.5	
Cognitive Battery		
3MSE	90.9 (7.1)	92 (43–100)
DSST	50 (16)	50 (10–106)
RAVLT‐R	41.2 (10.2)	41 (11–66)
RAVLT‐RI	7.64 (3.2)	8 (0–15)
COWA‐Semantic	30.8 (8.4)	30 (11–69)
COWA‐Phonemic	29.7 (11.7)	29 (2–67)
Stroop word card	19.2 (5.1)	18 (11–75)
Stroop color card	26 (7.1)	25 (14–88)
Stroop color‐word card	59.2 (22.2)	53 (26–193)

3MSE, Modified Mini‐Mental State Examination; BMI, body‐mass index; COWA, Controlled Oral Word Association Task; CVD, cardiovascular disease; DSST, digit symbol substitution Test; RAVLT, Rey Auditory‐verbal Learning Task; R, recall; RI, retroactive interference.

All studies were approved by the Wake Forest School of Medicine Institutional Review Board and conducted in accordance with the Declaration of Helsinki. All experiments were undertaken with the understanding and written consent of subjects.

### Cognitive tasks

This study includes an extension and secondary analysis of cognitive battery data described in detail in a previous study (Hugenschmidt et al. 2013). The battery assessed a number of cognitive domains by employing the Modified Mini‐Mental State Examination (3MSE; *n* = 883); the DSST (Digit Symbol Substitution Task; *n* = 889); the Rey Auditory‐Verbal Learning Task (RAVLT‐R; *n* = 891); the COWA (Controlled Oral Word Association Task; *n* = 849); and the Stroop Task (*n* = 883). The 3MSE is often used to screen for cognitive impairment and dementia; a series of questions were used to assess an individual's orientation to place and time, word recall and fluency, and construction (McDowell et al. 1997). The DSST measured short‐term working memory by asking individuals to match symbols to numbers as quickly as possible within a limited amount of time (Swiger et al. 2013). The RAVLT measured episodic memory and verbal learning by reading individuals a set of 15 unrelated nouns (known as list A) and asking them to repeat back as many as they could remember. Scores are the sum of correct words recalled across the first five trials. A subsection of the test required individuals to recall words from list A, directly after the introduction of a distractor list B. A measure of performance during this subsection is termed retroactive interference (RAVLT‐RI) – as recently learned words from list B interfere with the recall of words from list A (Mitrushina et al. 1991). The COWA task was used to measure phonemic (letter) and semantic (category) fluency by allotting individuals one minute to name as many words as possible starting with a given letter (F,A,S) or within a given category (animals and kitchen) (Shao et al. 2014). The Stroop task was broken down to three phases. First, individuals were shown a word card (W) and asked to read the names of colors printed in black ink. Then they were shown a color card (C) and asked to name color swatches. Finally, individuals were presented a CW (color‐word) card and asked to name the incongruent color of the print used for the color names, while trying to suppress the automatic processing of printed words. For the Stroop analysis, a group of subscores (Table S1) was chosen based on previous analyses and including two measures of the stroop effect (Jensen 1965). Color‐blind individuals were excluded from the Stroop Task (*n* = 2). Importantly, this study aimed to examine the functional effects of dopaminergic polymorphisms in a T2D‐affected population on cognitive performance, thus individuals whose performance was indicative of mild cognitive impairment or dementia were not excluded. Scores were natural log transformed when necessary to approximate a normal distribution.

### Genotyping

Initially, SNPs (single‐nucleotide polymorphisms) were selected to form an a priori set of candidate variants covering genes in the dopaminergic pathway, the insulin signaling pathway, and the convergence of both. Genotyping was accomplished using the Sequenom MassARRAY iPLEX^™^ multiplexing assay. In general, the protocol included a multiplex polymerase chain reaction (PCR) followed by a single‐base primer extension reaction and MALDI‐TOF mass spectrometry‐based allele detection (Oeth et al. 2005). During quality control, both samples and SNPs were excluded with call rates less than 90% and discordant samples were removed. The final candidate gene list included 13 SNPs (Table 2).

**Table 2:** Candidate variants, supporting literature, and association *P*‐values (*β* –values) with cognitive phenotypes assuming an additive model of inheritance

Chr	Position	Rsid	Gene	Name	Function	Location	Alleles mjr/mnr	MAF	Association *P*‐values (*β*‐values); covariates – age, sex, T2D affected status, education^a^										
									3MSE	DSST	RAVLT‐R	RAVLT‐RI	COWA‐ Semantic	COWA‐ Phonemic	Stroop‐I	Stroop‐II	Stroop‐III	Stroop‐IV	Rationale
5	13,92,904	rs28363170	DAT1	Dopamine Active Transporter 1	Membrane spanning receptor; mediates reuptake of dopamine from synapse	3' UTR	10‐R/8, 9, 11‐R	0.28^b^	0.616 (−0.182)	0.866 (−0.132)	0.835 (0.105)	0.400 (−0.129)	0.671 (0.192)	0.403 (0.525)	0.860 (−0.004)	0.337 (0.040)	0.470 (l−0.019)	0.253 (0.029)	Newman et al. (2014), Schneider et al. (2015), Yang et al. (2007)
14	10,47,93,397	rs1130214	AKT1	v‐akt murine thymoma viral oncogene homolog 1	serine‐threonine protein kinase involved in metabolism and DA receptor signaling	3' UTR	G/T	0.24	0.711 (0.134)	0.377 (0.687)	0.256 (0.563)	0.084 (0.256)	0.254 (−0.516)	0.828 (−0.138)	0.777 (−0.006)	0.418 (0.032)	0.153 (−0.037)	**0.004 (0.070)**	Cox et al. (2014), Mackenzie and Elliott (2014), Xia et al., 2015
4	11,34,58,223	rs1880529	CAMKIID	Calcium/Calmodulin‐ Dependent Protein Kinase II Delta	Regulates calmodulin‐dependent protein kinase activity; positive regulation of ERK signaling pathway	Intronic	T/C	0.32	0.514 (0.225)	0.666 (−0.320)	0.516 (−0.309)	0.570 (0.082)	0.355 (−0.400)	0.463 (−0.443)	0.493 (−0.015)	0.870 (−0.006)	0.926 (−0.002)	0.862 (−0.004)	Sprooten et al. (2013), Southam et al. (1999), Zhang et al. (2014)
7	5,05,50,906	rs10499695	DDC	DOPA decarboxylase	Catalyzes decarboxylation of L‐DOPA to DA	Intronic	T/C	0.46	**0.020 (**−**0.761)**	0.223 (−0.855)	**0.039 (**−**0.928)**	**0.001 (**−**0.448)**	0.751 (−0.130)	0.898 (0.073)	0.177 (−0.027)	0.503 (−0.025)	0.333 (−0.023)	0.969 (−0.001)	Borelli et al. (1997), Rorsman et al. (1995), Zhu et al., (2013)
7	5,04,77,395	rs3887825	DDC	DOPA decarboxylase	Catalyzes decarboxylation of L‐DOPA to DA	Intronic	G/A	0.47	0.228 (0.411)	0.806 (0.180)	0.155 (0.670)	0.375 (0.126)	0.889 (0.059)	0.938 (−0.046)	0.773 (−0.006)	0.602 (0.020)	0.790 (−0.007)	0.598 (0.012)	Borelli et al. (1997), Rorsman et al. (1995) Zhu et al., (2013)
7	5,05,56,808	rs6969081	DDC	DOPA decarboxylase	Catalyzes decarboxylation of L‐DOPA to DA	Intronic	T/A	0.5	0.217 (−0.431)	0.915 (−0.080)	0.277 (−0.524)	**0.010 (**−**0.376)**	0.794 (−0.114)	0.425 (0.487)	0.379 (−0.019)	0.996 (0.0002)	0.429 (−0.020)	0.139 (−0.035)	Borelli et al. (1997), Rorsman et al. (1995), Zhu et al., (2013)
3	11,41,71,968	rs6280	DRD3	Dopamine Receptor D3	DA receptor localized to limbic regions; inhibits AC	Exonic	T/C	0.49	0.475 (0.240)	0.853 (0.133)	0.230 (0.554)	0.373 (0.124)	0.249 (−0.484)	0.616 (−0.295)	0.982 (−0.0004)	0.796 (0.010)	0.990 (0.0003)	0.472 (0.016)	Bombin et al. (2008), Ustione and Piston (2012)
10	6,07,92,132	rs2456778	FAM53B	Family With Sequence Similarity 53, Member B	Regulates *β*‐catenin nuclear localization	Intronic	T/A	0.21	0.951 (0.023)	0.571 (0.465)	0.653 (0.238)	0.0862 (−0.277)	0.067 (0.875)	0.301 (0.692)	0.983 (0.0005)	0.826 (−0.010)	0.719 (−0.010)	0.289 (0.028)	Gelernter et al. (2014), Kanazawa et al. (2005), Kizil et al. (2014)
11	28,18,521	rs2237892	KCNQ1	Potassium Channel, Voltage Gated KQT‐ Like Subfamily Q, Member 1	Action potential repolarization	Intronic	C/T	0.15	0.704 (0.284)	0.492 (−1.10)	0.453 (−0.771)	0.758 (−0.096)	0.697 (0.361)	0.973 (0.044)	0.271 (0.051)	**0.024 (**−**0.189)**	0.089 (0.093)	0.799 (0.013)	Abbott (2014a), Abbott et al. (2014b), Hansen et al. (2006) Thevenod (2002), Unoki et al. (2008)
11	28,35,964	rs2237895	KCNQ1	Potassium Channel, Voltage Gated KQT‐ Like Subfamily Q, Member 1	Action potential repolarization	Intronic	A/C	0.32	0.177 (0.447)	**0.018 (1.67)**	0.398 (−0.384)	0.371 (0.124)	0.214 (0.509)	0.190 (0.751)	0.556 (−0.012)	0.394 (−0.032)	0.736 (−0.008)	0.958 (0.001)	Abbott (2014a), Abbott et al. (2014b), Hansen et al. (2006) Thevenod (2002), Unoki et al. (2008)
11	28,28,300	rs2283228	KCNQ1	Potassium Channel, Voltage Gated KQT‐ Like Subfamily Q, Member 1	Action potential repolarization	Intronic	A/C	0.16	0.243 (0.810)	0.910 (0.171)	0.927 (−0.088)	0.848 (0.055)	0.183 (1.15)	0.581 (0.668)	0.889 (−0.006)	0.149 (−0.112)	0.806 (0.012)	0.621 (−0.023)	Abbott (2014a), Abbott et al. (2014b), Hansen et al. (2006), Thevenod (2002), Unoki et al. (2008)
11	9,29,75,544	rs1080963	MTNR1B	Melatonin Receptor 1B	Implicated in circadian entrainment and GPCR signaling	Intronic	C/G	0.26	0.723 (−0.125)	0.302 (−0.784)	0.071 (−0.878)	0.187 (−0.194)	0.684 (−0.178)	0.459 (−0.454)	0.880 (0.003)	**0.038 (**−**0.082)**	0.293 (0.027)	0.053 (0.046)	Bonnefond and Froguel (2015), Yokoyama et al. (2014)
1	1,12,62,099	rs1883965	MTOR	Mechanistic Target Of Rapamycin	Kinase; mediates cellular response to stress	Intronic	G/A	0.31	0.251 (−0.429)	**0.045 (**−**1.59)**	0.489 (−0.359)	0.268 (−0.173)	**0.025 (**−**1.03)**	0.764 (−0.193)	0.516 (0.014)	0.845 (0.008)	0.506 (0.018)	0.993 (−0.0002)	Bowling et al. (2014), Melnik (2015), Johnson et al. (2013)
11	21,72,610	rs10770141	TH	Tyrosine Hydroxylase	Rate‐limiting enzyme in the synthesis of DA	Upstream	G/A	0.34	0.706 (0.116)	0.184 (−0.871)	0.634 (−0.202)	0.568 (−0.074)	0.433 (−0.302)	0.249 (−0.621)	0.521 (−0.012)	0.682 (−0.014)	0.470 (−0.016)	0.242 (0.025)	Aliev et al. (2014), Borelli et al. (2003), Rao et al. (2010)

AC, adenylyl cyclase; DA, dopamine; ERK, extracellular signal regulated kinases; GPCR, G‐protein coupled receptor; K(+), potassium; L‐DOPA, L‐3,4‐dihydroxyphenylalanine.

Association *P*‐value <0.05 in bold.

a RAVLT‐RI additionally adjusted for number of words recalled after initial exposure.

b MAF based on frequency of all minor alleles.

For the *DAT1* VNTR, genotyping was achieved using PCR with the forward primer: 5'‐ TGTGG TGTAGGGAACGGCCTGAG‐3' and reverse primer: 5'‐CTTCC TGGAGGTCACGGCTCAAGG‐3' followed by 2% agarose gel electrophoresis. Allele determination was made based on the size of fragments compared with known genotypes (Sano et al. 1993; Kang et al. 1999) and standards.

Finally, SNPs in loci of interest with strong evidence for involvement in DA processing (*DDC*,* TH*,* DRD2*,* DRD3*, and *DAT1*) were extracted from genetic datasets available in a subset of the cohort (*n* = 572) derived from Affymetrix^®^ Genome‐Wide Human SNP Array 5.0 and 1000 Genomes (1000G) imputation from this array. The genes included were *DAT1* (*SLC6A3*, DA transporter), *DDC* (DOPA decarboxylase), *DRD2* (DA D2 receptor), *DRD3* (DA D3 receptor), and *TH* (tyrosine hydroxylase). Genotyping and quality control procedures for these data are described elsewhere (Cox et al. 2014). Briefly, for the GWAS set, exclusion criteria for SNP performance included Hardy–Weinberg equilibrium *P* < 1 × 10^−6^, call rate <95%, and minor allele frequency <0.01. Only SNPs with a confidence score >0.90 and information score >0.50 were used from the imputed dataset. Initially 649 SNPs were isolated from both datasets. After reduction due to >5% missingness and <5% minor allele frequency, 484 SNPs remained for analysis.

### Statistical analyses

To determine the effect of candidate variants on cognitive performance, genetic association analyses were completed for the 13 candidate SNPs and the *DAT1* VNTR. Next, targeted association analyses were performed using the GWAS and imputed data. All SNP association analyses were carried out using variance‐components analysis in SOLAR version 8.0.1 (Texas Biomedical Research Institute San Antonio, TX) to account for relatedness among subjects (Almasy and Blangero 1998). Genetic association was investigated assuming an additive model of inheritance and adjusting for age, sex, T2D‐affected status, and education. An additional covariate was added when analyzing retroactive interference in the RAVLT in order to adjust for recall performance after a single exposure to the word list. Significance levels were adjusted for multiple comparisons by Bonferroni correction. For the candidate variant analysis, *P *<* *3.8 × 10^−3^; for SNPs extracted from genome‐wide and imputed data, statistical significance was accepted at 1 × 10^−4^.

## Results

Table 1 provides summary statistics of the analyzed sample. The significant enrichment of T2D‐affected participants is reflected in prevalent comorbid indices such as participant‐reported CVD (cardiovascular disease; 34.8%), high mean BMI (body‐mass index; 32.5 kg/m^2^), and a significant proportion of individuals with hypertension (86.9%). In general, cognitive performance among the different tests was normally distributed and performance ranged from above average to indicative of cognitive impairment (Hoyer et al. 2004; Loonstra et al. 2001; Savage and Gouvier 1992; Roy et al. 2015; Vogel et al. 2013; Table 1). Education levels ranged from less than high school to attainment of highly trained occupations which is reflective of a community‐based cohort.

Initially, the cognitive data from examination of these subjects were evaluated for association with 14 genetic variants. Characteristics of candidate SNPs and *DAT1* VNTR are summarized in Table 2 with a brief description of gene function and relevant references. Table 2 also summarizes the results of the genetic association analysis. The variant analysis was conducted in the DHS – Mind cohort to evaluate the association of dopaminergic and other literature‐supported polymorphisms with cognitive performance adjusting for age, sex, T2D status, and education

Assuming an additive model with the 10 repeat allele as the risk allele (Yang et al. 2007), the *DAT1* VNTR was not significantly associated with any cognitive phenotypes (Table 2). The most suggestive association was with poorer performance (i.e., higher score) on the Stroop‐IV, which measures difficulty in color‐naming due to interference of the conflicting printed words known as the stroop effect (*P *=* *0.253; *β *= 0.029).

In general, when SNPs in candidate genes were evaluated (Table 2) most associations were, at best, nominal. However, one coding variant from *DDC*, the DOPA decarboxylase gene, rs10499695 was significantly associated with poorer performance on a subset of RAVLT that measures retroactive interference (*P* = 0.001, *β *= −0.45). Additionally, an *AKT1* gene polymorphism, rs1130214, was nominally associated with performance on Stroop‐IV (*P *=* *0.004; *β *= 0.07), though this did not meet our Bonferroni‐corrected *P*‐value threshold.

**Figure 1:**
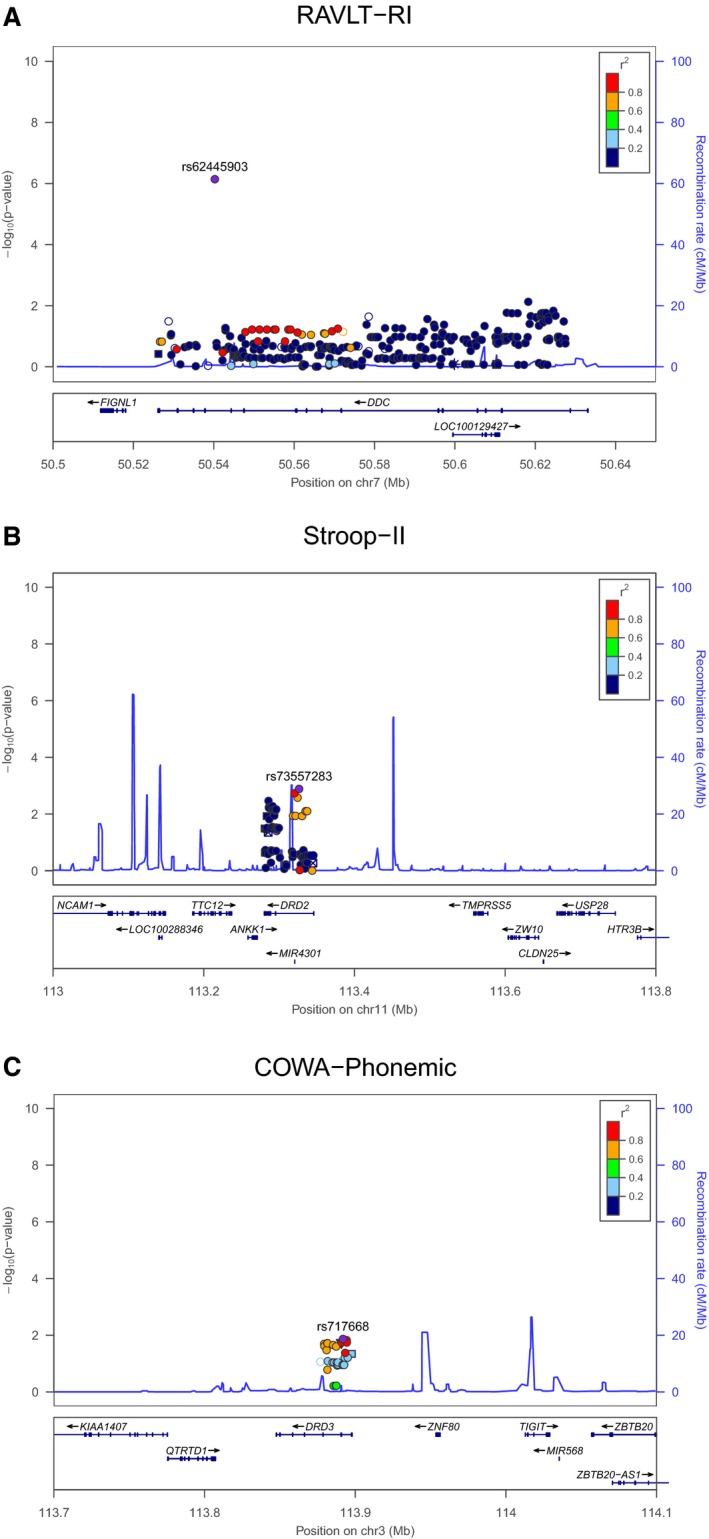
Locus plots for GWAS and Imputed associations with (A) RAVLT‐RI (B) Stroop‐II and (C) Stroop‐IV. Association analyses were performed assuming an additive model of inheritance with adjustment for age, sex, T2D affected status, and education; additional adjustment for recall performance after first exposure to the word list was included for RAVLT‐RI. Abbreviations: GWAS, genome‐wide association study; RAVLT‐RI, Rey Auditory‐verbal Learning Task—retroactive interference; T2D, type 2 diabetes.

Taking advantage of GWAS and imputed data, a targeted analysis was carried out in a subset of the cohort (*n* = 572). 484 SNPs in our genes of interest from GWAS and imputed data passed quality control. The top 50 SNPs associated with each of the cognitive phenotypes are included in Table S2A–J. This targeted association analysis revealed a significant association between SNP rs62445903 in *DDC* and retroactive interference on the RAVLT (*P *=* *7.21 × 10^−7^, *β *= 0.3). Among the most strongly associated SNPs were several variants from *DRD2* (e.g., rs73557283, *P *=* *1.3 × 10^−3^, *β *= 0.12 and rs77195172, *P *=* *1.85 × 10^−3^, *β *= 0.12 with Stroop‐II score) and *DAT1* (e.g., rs10052016, *P *=* *1.61 × 10^−3^, *β *= 0.11 and rs10053602, *P *=* *3.94 × 10^−3^, *β *= 0.10 with Stroop‐IV score) genes. Figure 1 represents locus plots of the DOPA decarboxylase, DA D2 receptor, and DA D3 receptor genes in RAVLT‐RI, Stroop‐II, and COWA‐Phonemic tasks, respectively.

## Discussion

After searching the literature, candidate genetic variants were selected based on studies investigating specific components of the dopaminergic pathway, cognitive function, T2D pathogenesis, and particularly combinations thereof. In the T2D‐enriched DHS sample, the majority of candidate SNPs were not strongly associated with measures of cognitive function at conservative levels of significance. However, of special interest is SNP rs10499695 located intronically in the gene *DDC*, (DOPA decarboxylase) which was significantly associated with retroactive interference on the RAVLT. In this section of the RAVLT task, a distractor list is read between presentation and recall of the original word list; disruption of recall of the retained words is termed retroactive interference. Interestingly, this *DDC* SNP has previously been shown to be associated with alerting attention, which specifically measures an individual's ability to maintain a state of alertness and readiness to respond to stimuli (Zhu et al. 2013). This is congruent considering it is theoretically a diverted attention, e.g., the distractor list, which underpins the phenomena of retroactive interference (Dewar et al. 2007).

An extensive candidate gene literature surrounds the *DAT1* VNTR, a 40‐base pair repeat unit with 9 and 10‐repeat units occurring most commonly. For example, Schneider and colleagues found that 10‐repeat carriers were less susceptible to the “Stroop effect” (2015) that arises from discordance between words and color words, and is scored similar to Stroop‐III in this study. However, *DAT1* genotype was not associated with Stroop‐III performance or any other cognitive measure in the DHS – Mind sample. This is not surprising given that replication in both genetic and cognitive literature is a challenge due to variations in sample size, misinterpretation of associations (Hirschhorn 2005), and heterogeneity of phenotyping procedures (e.g., see Hart et al. 2013). However, the importance of the *DAT1* VNTR should not be discarded in light of a recent meta‐analysis of in vivo imaging studies which found that *DAT1* VNTR genotype has functional consequences on DA transporter activity (Faraone et al. 2014). Thus, future studies are needed to completely understand the role of the *DAT1* VNTR in cognitive performance in T2D‐affected individuals.

Next, to explore the genetic consequence of the dopaminergic system on cognitive capacity in T2D, association analyses of both genotyped and imputed SNPs from regions of interest were completed. The most notable finding was that a significant association between a polymorphism from the DOPA decarboxylase gene and retroactive interference in the RAVLT was revealed. This intronic variant, rs62445903, is located approximately 78 kb upstream of the significant *DDC* SNP, rs10499695, uncovered in the candidate gene analysis. A pair‐wise linkage disequilibrium test using 1000G as the reference panel suggested the two SNPs from *DDC* are most likely in linkage disequilibrium, with a reduced correlation coefficient due to a MAF less than 10% for the imputed SNP, rs62445903 (*D*' = 1; *r* ^2^ = 0.12). Furthermore, rs62445903 is no longer significant when the model is adjusted for the candidate variant rs10499695. Although rs10499695 was also included on the Affymetrix^®^ array, it failed to reach significance in this secondary analysis. This could be due to the reduced sample size in the GWAS and imputed analysis compared to the candidate SNP analysis (*n* = 572 vs. *n* = 893, respectively) especially since the two genotyping procedures were 99.1% concordant. Importantly, the appearance of significant SNPs on the *DDC* locus in both analyses highlights this gene as a locus of interest for cognitive capacity in T2D.

Interestingly, the GWAS/Imputation analysis revealed DOPA decarboxylase polymorphisms among the 50 strongest associations for each of the cognitive indices. The *DDC* gene undergoes alternative splicing creating two distinct mRNAs – neural and nonneuronal (Ichinose et al. 1992). Although it is most well‐known for synthesis of DA and serotonin in the central nervous system, the enzyme is also located in pancreatic *β* cells where it is theorized to be involved in local, dopaminergic regulation of insulin secretion (Rubi et al. 2005; Ustione et al. 2013). The abundance of nominal associations in the *DDC* gene in a T2D population has biological relevance with regard to potential roles in cognitive function as DDC deficiency due to frame‐shift mutations or substitutions leads to impaired cognitive function (Bertoldi 2014).

Intriguingly, the most significant SNP associations in the GWAS and imputation analysis (refer to Table S2) were generally segregated by cognitive phenotype. For instance, 100% of the top 50 SNPs associated with Stroop‐I score were from *DDC*, while 43 of the top 50 associations for Stroop‐II score were polymorphisms from the *DRD2* gene. This is compelling since Stroop‐I scores represent an individual's personal tempo, while Stroop‐II scores represent a color‐naming factor (Jensen 1965). Thus, this study tenably uncovered a genetic component that further validates Jensen's statistical conclusion of a cluster of Stroop subscores representing different cognitive dimensions (1965).

Although the present analysis was able to reveal polymorphisms significantly and suggestively associated with cognitive phenotypes in a T2D‐rich familial population, there are limitations to the study. Firstly, as with the majority of studies investigating the genetic contribution to complex disorders, sample size is a concern; the GWAS and imputed association analysis in particular was limited to fewer than 500 individuals (Hong and Park, 2012). Thus, a future study closely investigating the influence of the dopaminergic pathway on cognition in a larger cohort is warranted. Secondly, candidate genes for this study were selected by gathering information from previous genetic studies and knowledge of biologic pathways in the pursuit of SNPs that harbor functional significance. However, the majority of the candidate SNPs as well as the current significant associations included intronic variants. Intronic SNPs may act as regulatory elements through interaction with enhancers to affect transcription (Stadhouders et al. 2012), or may affect chromatin structure by changes in DNA methylation patterns, thus modulating gene activity (Zaina et al. 2010). SNP rs10499695 is associated with retroactive interference and is located within an ETS‐family transcription factor binding site (Boyle et al. 2012). Moreover, these variants may not be protein‐coding but the field of medical genetics requires a more thorough understanding of polymorphisms outside protein‐coding regions, especially for those that may affect RNA splicing (Xiong et al. 2015). For instance, the other *DDC* SNP significantly associated with retroactive interference, rs62445903, is located in intron 9. Interestingly, there is an isoform of human *DDC* that includes an alternative exon 10 that is located within intron 9 and this particular isoform is expressed in nonneural kidney tissue (Vassilacopoulou et al. 2004). Effects on alternative splicing are one way intronic SNPs modulate mRNA expression and translation and this is perhaps the mechanism of action by which *DDC* affects cognition. Finally, it is possible that the DDC SNPs tagged in this study may be in LD with untyped putatively functional variants, which was not investigated in this study.

In conclusion, this study offers evidence of the involvement of dopaminergic genes in cognitive performance among T2D‐affected individuals, especially through regulation of DA synthesis. Identification of such pathways is critical in order to unveil new avenues of treatment as well as garner a more thorough understanding of the detrimental cognitive effects of T2D. Future studies will be needed to validate the findings and expand knowledge on the role of the *DDC* gene in individual cognitive ability.

## Conflict of Interest

None declared.

## Supporting information


**Table S1.** Formulas for derived Stroop scores.

**Table S2.** Top 50 association *P*‐values of genome‐wide and imputed data with cognitive phenotypes adjusted for age, sex, T2D‐affected status, and education. RAVLT‐RI model additionally adjusted for recall performance after first exposure to the word list. Abbreviations: RAVLT‐RI, Rey Auditory‐verbal Learning Task—retroactive interference; T2D, type 2 diabetes.
