# Supplementary material for: Dopamine pathway gene variants may modulate cognitive performance in the DHS – Mind Study
Source: Brain Behav. 2016 Mar 15;6(4):e00446. doi: 10.1002/brb3.446 (PMC4797918; doi:10.1002/brb3.446)
Supplement: Supplementary file 1 — Table S1. Formulas for derived Stroop scores. [file BRB3-6-e00446-s001.docx]

**Supplementary Table 1**.

| Subtest (measured in seconds to complete) | | | | | |
| --- | --- | --- | --- | --- | --- |
|  | W | Word card | |  | |
|  | C | Color card | |  | |
|  | CW | Color-word card | |  | |
|  | Derived scores | Scoring formulas | |  | |
|  | I | C |  | |  |
|  | II | W/(W+C) |  | |  |
|  | III | CW - W |  | |  |
|  | IV | W/CW |  | |  |
|  |  |  |  | |  |

Formulas for derived Stroop scores
